# Serum response factor is overexpressed in esophageal squamous cell carcinoma and promotes Eca-109 cell proliferation and invasion

**DOI:** 10.3892/ol.2013.1120

**Published:** 2013-01-09

**Authors:** XI HE, HONG XU, MIN ZHAO, SHIJIE WANG

**Affiliations:** 1 Department of Surgery, Hebei Medical University Research Center, The Fourth Affiliated Hospital of Hebei Medical University, Shijiazhuang, Hebei 050011;; 2 Medical Research Center, Hebei United University, Tangshan, Hebei 063000;; 3 Department of Pathology, The Qinhuangdao First Hospital, Qinhuangdao, Hebei 066000, P.R. China

**Keywords:** serum response factor, E-cadherin, β-catenin, esophageal squamous cell carcinoma, cyclin D1, Eca-109

## Abstract

Recent studies indicate that serum response factor (SRF) is highly expressed in tumors such as hepatocellular, thyroid, esophageal and lung carcinoma. However, the expression and roles of SRF in esophageal squamous cell carcinoma (ESCC) are unclear. In this study, immunohistochemistry was used to compare the expression of SRF in ESCC cases (n=73) and normal controls (n=30). The RNA interference (RNAi) technique was used to knock down the expression of SRF in Eca-109 cells. Cell proliferation, cell cycle stage and invasion were measured with cell counting kit (CCK)-8, flow cytometry and Transwell assays, respectively. Western blotting was used to measure SRF, E-cadherin, β-catenin and cyclin D1 expression in Eca-109 cells treated with siRNA. The study demonstrated that human ESCC has increased expression of SRF. In addition, blocking SRF expression inhibited tumor proliferation and invasion. In conclusion, SRF has the potential to be a new marker for ESCC diagnosis and therapy.

## Introduction

Esophageal cancer is the sixth leading cause of cancer-related mortality worldwide, but it is also the least studied type of tumor. There is an exceedingly high incidence of esophageal squamous cell carcinoma (ESCC) in Asian countries, particularly in north and central China.

Although 90% of cancer mortalities are caused by metastasis, the mechanisms of metastasis remain poorly defined. Consequently, a better understanding of metastasis offers promise for the development of improved cancer therapies (1–3). Deterioration of cell-cell and cell-extracellular matrix (ECM) adhesions is often observed in tumor cells, and this may be associated with the invasion and metastasis of cells into surrounding tissues and blood vessels. Epithelial cadherin (E-cadherin) is thought to mediate cell-cell adhesion, and this protein plays a critical role in cancer invasion and metastasis. E-cadherin complexes with other submembraneous cytosolic proteins, including E-catenin and β-catenin, and these catenins mediate the connection of E-cadherin to actin filaments. Altered expression of the E-cadherin/β-catenin complex is associated with de-differentiation, invasion and metastasis of tumors (4).

Serum response factor (SRF) is a member of the highly conserved MADS (MCM1, Agamous, Deficiens, SRF) box family of transcription factors which regulates the expression of immediate early genes, such as *c-fos*, muscle-specific genes, and genes involved in cytoskeleton regulation, motility and adhesion (5). A number of researchers have reported that SRF is highly expressed in tumors, including colorectal cancer (6), hepatocelluar carcinoma (7,8), breast cancer (5) and thyroid carcinoma (9). Overexpression of SRF in hepatocellular carcinoma and breast cancer accelerates cell migration and invasion, and there is a subsequent acquisition of mesenchymal phenotypes due to the expression of a mesenchymal marker (vimentin) (5,10). Furthermore, overexpression of SRF in colorectal cancer has been reported to decrease E-cadherin expression and increase nuclear β-catenin expression (6).

Expression of SRF in ESCC and its role in the modulation of the E-cadherin/β-catenin complex have not been investigated. In this study, we examined the expression of SRF in ESCC. We also examined the effect of the downregulation of SRF by RNA interference (RNAi) on the proliferation and invasion of Eca-109 cells via altered expression of SRF, E-cadherin and β-catenin.

## Materials and methods

### Tissue sample collection

We retrospectively studied ESCC specimens from surgical resections taken between 2009 and 2011 at Tangshan People’s Hospital, China. These patients (n=73) did not receive any preoperative adjuvant radiation or chemotherapy. All research involving human participants was approved in writing by the patients studied and the ethics committee at Hebei Medical University.

### Immunohistochemistry for SRF

Paraffin-embedded sections were permeabilized with 0.2% Triton and blocked with 5% bovine serum albumin (BSA) in 0.1 M phosphate-buffered saline (PBS) for 30 min to reduce nonspecific binding, followed by incubation with primary antibodies against SRF (sc-335; Santa Cruz Biotechnology, Inc., Santa Cruz, CA, USA), biotinylated secondary antibody and ABC reagent (Boshide Bio, Inc., Wuhan, China). Immunoreactivity was visualized with DAB. Staining was scored independently by two observers, and a high level of concordance (90%) was achieved. When the observers disagreed, the slides were reviewed to arrive at a consensus.

Clear nuclear SRF staining in tumor cells was defined as SRF-positive (6). For assessment of SRF proteins, two scores were assigned to each core: i) staining intensity was scored as 0 (absent), 1 (weak), 2 (moderate) or 3 (strong); and ii) the percentage of positively stained epithelial cells was scored as 0 (<10% positive), 1 (10–30%), 2 (31–70%) or 3 (>70%). An overall protein expression score was calculated by multiplying the intensity and positivity scores (overall score range, 0–9), and further simplified by dichotomization to negative (≤3) or positive (≥4).

### SRF silencing

Cells were transfected with small interfering RNA (siRNA) against SRF using the Lipofectamine 2000 transfection reagent according to the manufacturer’s instructions (Shanghai GenePharma Co., Ltd, China). siRNA with the following sequences were obtained from GenePharma: i) siRNA-SRF-1107: sense: 5′-GCAAGGCACUGAUUCAGA CTT-3′ and antisense: 5′-GUCUGAAUCAGUGCCUUG CTT-3′ (in our preliminary experiment, we found that siRNA-SRF 1107 had a higher effect than others measured by real-time PCR and western blot analysis); ii) Negative-siRNA: sense: 5′-UUCUCCGAACGUGUCACGUTT-3′ and antisense 5′-ACGUGACACGUUCGGAGAATT-3′. Briefly, 1×10^5^ human ESCC Eca-109 cells (Cell Resource Center, Shanghai Life Sciences Institute, Chinese Academy of Sciences) per well were plated in 6-well plates and cultured to reach 80% confluence. Cells were then transfected with siRNA using transfection reagent in serum-free medium.

### Cell lines and proliferation assay

Eca-109 cells were maintained in a 5% CO_2_ atmosphere at 37°C in Dulbecco’s modified Eagle’s medium (DMEM) containing 10% fetal bovine serum (FBS). The cells were divided into four groups: i) control: serum-free; ii) siRNA-SRF: siRNA-SRF+RNAi-mate; iii) negative control: siRNA-negative control+RNAi-mate; and iv) mock transfection: RNAi-mate. All experiments were performed in triplicate.

The Eca-109 cells were seeded in parallel into 96-well tissue culture plates at a density of 5×10^3^ cells per well in full growth medium (DMEM plus 10% FBS). Cells were incubated overnight, then quiesced in serum-free medium for 12 h before treatment with siRNA-SRF. After treatment for 48 h, the medium was removed and the cells were incubated with a 10% cell counting kit (CCK)-8 (Dojindo, Kumamoto, Japan) for 4 h at 37°C. Cells were counted with a microELISA plate reader at 450 nm.

### Cell cycle analysis

Cells were trypsinized, washed once with ice-cold PBS and fixed with 70% ethanol at −20°C overnight. After washing twice with PBS, cells were stained with 10 *μ*g/ml propidium iodide (Sigma, St. Louis, MO, USA) containing 1 mg/ml RNase A (Sigma) at 37°C for 20 min in the dark and analyzed with a FACSCalibur flow cytometer and CellQuest software.

### Cell invasion assays

Cell invasion assays were performed using a 24-well Transwell migration chamber (Corning Life Sciences, Acton, MA, USA). The upper and lower chambers were separated by a polyvinyl-pyrrolidone-free polycarbonate membrane with an 8-*μ*m pore size. Cells (4×10^4^ per well) were suspended in serum-free medium and placed in the upper chamber. Medium containing 2% FBS was used as the chemoattractant source. Twelve hours later, cells on the upper surface of the filter were wiped with a cotton swab. Cells on the lower surface of the filters were fixed and stained with Giemsa. Cells that migrated to the lower surface of the filter were counted under a light microscope at ×200 magnification in ten randomly selected fields per well.

### Western blot analysis

The total protein content of cells and lung tissue lysed by RIPA (ZO2338A, Aidlab; Beijing, China) was quantified with the BCA assay (PC0020, Solarbio; Beijing, China). Proteins (70 *μ*g/lane) were separated in 10% gel by SDS-PAGE and electrotransferred to a nitrocellulose membrane (Solarbio). Membranes were blocked with 5% non-fat milk and incubated overnight at 4°C with the primary antibody [anti-SRF; anti-E-cadherin (sc-7870); anti-β-catenin (sc-7870); anti-cyclin D1 (sc-8376); anti-β-actin (sc-47778); Santa Cruz Biotechnology] followed by alkaline phosphatase-conjugated secondary antibodies (E030220, E020210; Earthox, San Francisco, CA, USA). Target bands were visualized by the addition of BCIP/NET (E116; Amresco, Solon, OH, USA). Results were normalized with β-actin and expressed as the fold change from specific bands in the control group.

### Quantitative real time polymerase chain reaction (PCR) for SRF

The following oligonucleotide primers specific for human genes were used in this study: SRF, sense 5′-CTTAACATGGCATCTTCGACACT-3′ and antisense 5′-CTTAACCTCTAATCCCCATTGCT-3′; GAPDH, sense 5′-GGGAAACTGTGGCGTGAT-3′ and antisense 5′-TGGGTGTCGCTGTTGAAGT-3′. Total RNA was extracted from cells using TRIzol reagent (15596-026, Invitrogen Life Technologies, Carlsbad, CA, USA), and cDNA was generated from 1 *μ*g RNA using a random hexamer and the Omniscript RT kit (c28025-032, Invitrogen). Real-time PCR was performed as described in the PCR core kit of SBYR-Green (c11733-038, Invitrogen). The data were analyzed using the ΔΔCt method and presented as arbitrary units.

### Statistical analysis

Values are expressed as mean ± SEM. Comparisons between multiple independent groups were conducted using one-way ANOVA followed by post-hoc analysis with the Brown-Forsythe test and SPSS 13.0. P<0.05 was considered to indicate a statistically significant difference.

## Results

### SRF, E-cadherin and β-catenin protein expression in ESCC and lymph node metastatic foci and their clinical characteristics

**Figure 1:**
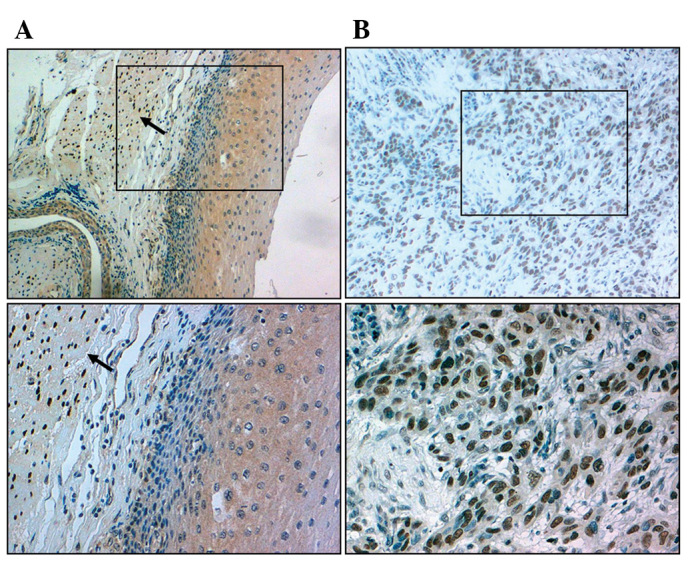
Evaluation of serum response factor (SRF) expression in esophageal squamous cell carcinoma (ESCC) and normal tissues (immunohistochemistry, ×200 and ×400). (A) The SRF-negative expression in normal esophageal tissue. The arrow shows SRF-positive expression in smooth muscle cell nuclei; (B) SRF-positive expression in esophageal carcinoma.

SRF protein positive detection rates in ESCC tissues (47.95%; 42/73) were higher than those of normal controls (20.00%; 6/30; χ^2^=12.037, P<0.05; Fig. 1). We also evaluated possible correlations between the expression of SRF and α-smooth muscle actin (α-SMA) in tumor cells with the clinicopathological characteristics of ESCC, including gender, age, tumor diameter, histological grade, lymph node metastasis and depth of invasion. SRF expression in the tumor cells was associated with poor differentiation, deep invasion and lymph node metastasis (Table I, P<0.05).

### siRNA-SRF-1107 reduces SRF mRNA and protein levels in Eca-109 cells

**Figure 2:**
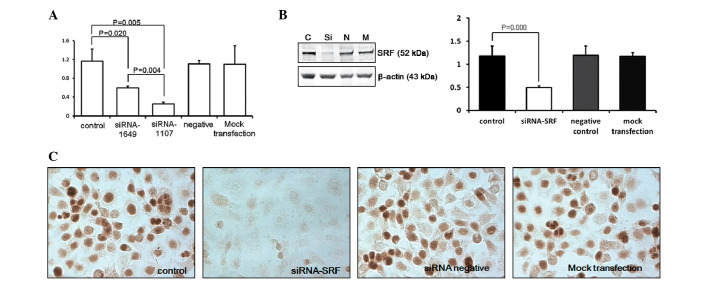
siRNA-SRF-1107 reduces serum response factor (SRF) mRNA and protein levels in Eca-109 cells. (A) mRNA expression of SRF in different groups; (B) SRF expression measured by western blot analysis; C, control; Si, small interfering RNA (siRNA)-1107; N, negative control; M, mock transfection; (C) SRF expression analyzed by immunocytochemistry (×400).

The ability of siRNA to reduce SRF mRNA and protein expression was analyzed using real-time PCR and western blot analysis, respectively. The expression levels of SRF mRNA in Eca-109 cells transfected with SRF-siRNA were reduced to 21.55% of those in the blank control group (P<0.01; Fig. 2A). SRF protein levels were reduced in Eca-109 cells transfected with SRF-siRNA to 41.53% of their levels in the blank controls (P<0.05). In addition, no difference between the blank control, negative siRNA control and mock transfection groups was observed (P>0.05; Fig. 2B and C).

### Effect of siRNA-SRF-1107 on proliferation of Eca-109 cells

**Figure 3:**
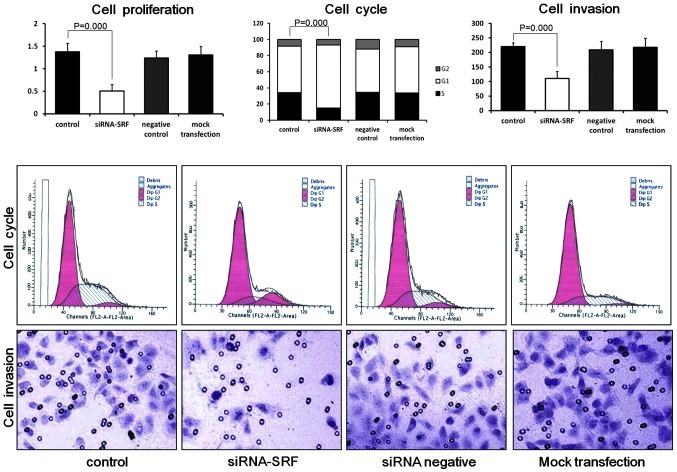
Effect of small interfering RNA-serum response factor (siRNA-SRF) on cell proliferation, cell cycle and cell invasion.

Cells in the four groups were harvested 48 h after transfection. The proliferation rate of the Eca-109 cells was significantly lower in the SRF-siRNA group than in the blank control (P<0.01), negative siRNA control (P<0.01) and mock transfection groups (P<0.01). No significant difference was observed between the blank control (P>0.05), negative siRNA control (P>0.05) and mock transfection groups (P>0.05; Fig. 3). These results suggest that the downregulation of SRF significantly inhibits the proliferation of Eca-109 cells.

### Downregulation of SRF affects cell cycle distribution in Eca-109 cells

The effect of SRF-siRNA on the cell cycle was evaluated by flow cytometry (Fig. 3). The four groups of cells were collected for cell cycle analysis 48 h after transfection. The percentage of S-phase cells in the siRNA-SRF-transfected group was lower than those for the blank control (P<0.05), negative siRNA control (P<0.05) and mock transfection groups (P<0.05). Therefore, SRF silencing may arrest the cell cycle at the G1 phase in Eca-109 cells.

### Effect of siRNA-SRF-1107 on invasion of Eca-109 cells

The invasive potential of Eca-109 cells was determined by using a Matrigel invasion assay (Fig. 3). Cells transfected with siRNA-SRF showed decreased migration (110.50±24.84) through the Matrigel compared with the blank control (220.17±12.94), negative siRNA control cell (217.67±31.26) and mock transfection groups (208.67±29.75; P<0.05). In addition, no difference between the blank control (P>0.05), negative siRNA control cell (P>0.05) and mock transfection groups (P>0.05) was observed. These results suggest that the downregulation of SRF significantly inhibits the invasive capacity of Eca-109 cells.

### Effect of siRNA-SRF-1107 on E-cadherin, β-catenin and cyclin D1 expression in Eca-109 cells

**Figure 4:**
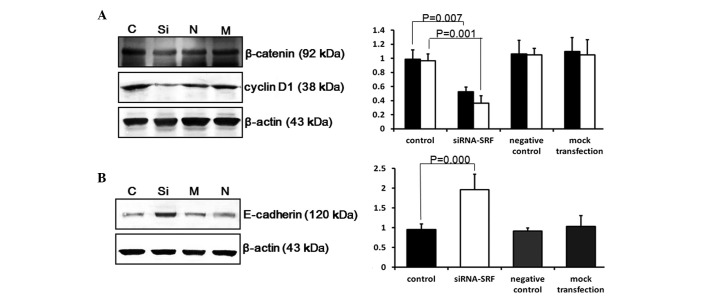
Effect of siRNA-SRF on expression of serum response factor (SRF), E-cadherin, β-catenin and cyclin D1 in Eca-109 cells. (A) β-catenin and cyclin D1 expression measured by western blot analysis; (B) SRF expression measured by western blot analysis; C, control; Si, small interfering RNA (siRNA)-1107; N, negative control; M, mock transfection.

Western blot analysis revealed that siRNA-SRF treatment of Eca-109 cells resulted in down-regulation of β-catenin and cyclin D1 protein expression by 52.53 and 38.14%, as compared with blank controls (Fig. 4A). Furthermore, gene silencing by siRNA-SRF-1107 markedly upregulated the E-cadherin expression 2.03-fold, compared with the control group (Fig. 4B). In addition, no difference between the blank control (P>0.05), negative siRNA control cell (P>0.05) and mock transfection groups (P>0.05) was observed.

## Discussion

Our results showed that SRF was more highly expressed in ESCC than normal esophageal tissue and that SRF levels were correlated with patient clinical parameters. We subsequently evaluated SRF function in Eca-109, an ESCC cell line. Knockdown of SRF in Eca-109 cells inhibited cell proliferation and invasion *in vitro*. These results suggest that SRF is involved in the development and progression of ESCC.

Serum response factor has been reported to be involved in promoting the carcinogenesis and progression of colorectal cancer (6), hepatocelluar carcinoma (7,8,10,11), breast cancer (5) and thyroid carcinoma (9). However, the role of SRF in ESCC and its mechanism of action have not been reported. The overexpression of SRF in cancer has increasingly been shown to enhance invasion and migration of cancer cells, due to loss of cell-cell adhesion (6), acceleration of cell migration and invasion in hepatocellular carcinoma, and acquisition of mesenchymal phenotypes due to the expression of a mesenchymal marker (vimentin) and the activation of immediate early genes (10,11). High SRF levels in carcinomas also contribute to ECM degradation and progressive tumor cell migration and invasion (8,12).

The current study characterizes SRF as a tumorigenic enhancer that regulates β-catenin and cyclin D1. β-catenin is an important mediator in the Wnt signaling pathway, and when activated, is translocated into the nuclei where it stimulates the transcription of target genes involved in cell proliferation (13). Cyclin D1 is a major transcriptional target of β-catenin signals that promotes G1/S transition in the cell cycle (14). We found that downregulation of SRF decreased β-catenin and cyclin D1 levels and this correlated with inhibition of cell proliferation and cell cycle arrest. The association of SRF inhibition with decreased levels of β-catenin and cyclin D1 that we identified may be relevant since β-catenin signaling is strongly linked to ESCC (15–17). Furthermore, we found that SRF upregulation in ESCC is associated with poor differentiation, deep invasion and lymph node metastasis. Therefore, SRF may enhance the metastatic capability of tumor cells. Consequently, SRF may be a risk factor for ESCC metastasis. We also found that SRF gene silencing strongly inhibits the cellular invasion that accompanies the upregulation of E-cadherin. Consequently, inhibiting the expression of E-cadherin blocks its activity in cell-cell adhesion, cancer invasion and metastasis (18).

In summary, our study demonstrated that ESCC had increased expression levels of SRF as well as altered expression levels of E-cadherin and β-catenin. Blocking SRF expression inhibited the proliferation and invasion of cancerous cells.

## Figures and Tables

**Table I:** Serum response factor (SRF) expression in relation to clinicopathological features in esophageal squamous cell carcinoma.

Clinicopathological features		SRF					α-SMA				
	n	+	−	%	χ^2^	P-value	+	−	%	χ^2^	P-value
Gender											
Male	55	31	24	56.36	0.125	0.724	25	30	54.55	0.554	0.457
Female	18	11	7	61.11			10	8	55.56		
Age (years)											
≥60	47	28	19	59.57	0.225	0.635	26	21	55.32	2.875	0.090
<60	26	14	12	53.85			9	17	34.62		
Diameter											
≥5 cm	40	25	15	62.50	0.893	0.345	22	18	55.00	1.765	0.184
<5 cm	33	17	16	51.51			13	20	39.39		
Differentiation											
High+moderate	22	8	14	36.36	5.777	0.016	12	10	54.55	0.550	0.485
Low	51	34	17	66.67			23	28	45.10		
Depth											
≤ Muscular layer	25	7	18	28.00	13.574	0.000	7	18	28.00	6.060	0.014
≥ Adventitia	48	35	13	72.92			28	20	58.33		
Lymphatic metastasis											
Positive	42	30	12	71.43	7.815	0.005	25	17	59.52	5.313	0.021
Negative	31	12	19	38.71			10	21	32.26		

α-SMA, α-smooth muscle actin.
